# Breaking barriers in health surveillance: privacy-preserving techniques for tracking vaccine hesitancy and disease outbreaks in Pakistan

**DOI:** 10.3389/fpubh.2026.1854347

**Published:** 2026-08-27

**Authors:** Muhammad Imran Shahid, Han Wanqu, Faiza Shah, Kunpeng Ai

**Affiliations:** College of Political Science and Public Administration, Henan Normal University, Xinxiang, China

**Keywords:** outbreak underreporting, Pakistan, Punjab, randomized response technique, sensitive health data, vaccine hesitancy

## Abstract

**Background:**

Precise measurement of sensitive public health outcomes is often limited by underreporting, stigma, and social desirability bias. These challenges can affect estimates of COVID-19 infection and vaccine hesitancy, leading to incomplete evidence for surveillance and public health decision-making. This study examined the use of privacy-preserving stratified randomized response models (SRRMs) to improve the estimation of sensitive health information in a population-based setting.

**Methods:**

We conducted a cross-sectional quantitative survey in Punjab, Pakistan, during June-August 2021. A total of 1,200 participants were recruited using simple random sampling with replacement within a stratified design, with equal allocation to urban (*n* = 600) and rural (*n* = 600) populations. Privacy-preserving SRRM-I and SRRM-II procedures were applied to estimate underreported outbreak cases and vaccine hesitancy while reducing response bias related to confidentiality concerns. Estimates were compared with directly reported responses, and precision was assessed using the percentage relative efficiency (PRE).

**Results:**

The empirical estimates reflect the June–August 2021 survey period in Punjab, Pakistan. Estimated outbreak prevalence was higher than directly reported prevalence in both urban and rural populations, indicating underreporting of infection. In urban areas, directly reported COVID-19 cases (10.5%) were lower than privacy-preserving estimates obtained using SRRM-I and SRRM-II (15.3% and 17.4%). In rural areas, directly reported cases (13.7%) were also lower than the corresponding estimates (16.7% and 19.5%). Vaccine hesitancy estimates were also higher under the privacy-preserving procedures (26.3% reported vs. 27.4% and 27.2%), although the difference was considerably smaller than for outbreak cases. All PRE values exceeded 100, indicating improved efficiency relative to the benchmark models. These findings suggest that conventional self-reporting underestimates sensitive public health outcomes, particularly where disclosure concerns are present.

**Conclusion:**

Privacy-preserving SRRM frameworks can improve the estimation of sensitive public health outcomes, including outbreak underreporting and vaccine hesitancy. The advantage of the two-stage design was most pronounced for the more sensitive outcome, indicating that the truthful-reporting parameter should be matched to the perceived sensitivity of the outcome under study. In settings where respondents are reluctant to disclose health-related information, such methods can strengthen surveillance data quality and support more reliable public health planning and policy decisions.

## Highlights

- Privacy-preserving methods reveal higher estimates of outbreak prevalence and vaccine hesitancy compared with directly reported data in both urban and rural populations in Punjab, Pakistan.
- In urban areas, outbreak cases were underreported (10.5% reported vs. 15.3%–17.4% estimated), and rural areas showed a similar pattern (13.7% reported vs. 16.7%–19.5% estimated).
- At the outcome level, vaccine hesitancy was also higher under the privacy-preserving frameworks (26.3% reported vs. 27.4% under SRRM-I and 27.2% under SRRM-II), with a smaller difference than for outbreak cases.
- The results indicate that privacy-preserving techniques provide more informative population-level estimates, particularly in settings with concerns about disclosure.
- These findings highlight the need for privacy-enhancing data collection methods to improve public health surveillance, policy planning, and health service interventions.

## 1 Background

There are still several obstacles in the way of accurately measuring sensitive public health outcomes, mostly due to problems including item non-response, misreporting, and socially acceptable responses (1–3). When people are asked to divulge private information that causes shame, humiliation, or unfavorable consequences, these difficulties become very noticeable (4, 5). Such biases can seriously damage the credibility of the data used for public health surveillance, resource allocation, policy formulation, and service planning in the context of health services research, especially when it comes to estimates of infection rates, vaccine hesitancy, and other sensitive health behaviors (6–8). Data-collection techniques that protect respondent privacy while maintaining the accuracy and statistical efficiency required for solid, trustworthy results are urgently needed to address these concerns (9–11). Although conventional direct-questioning approaches are simple to implement, they produce biased estimates when respondents are reluctant to disclose sensitive health information (12–14).

To tackle the methodological issues in analyzing sensitive quantitative variables, substantial contributions have been made in improving randomized response techniques. Huang (12), Shahid and Hussain (15), and Hussain and Shahid (16) advanced unbiased estimation procedures, enhancing both additive and optional scrambling-based models. Kumar et al. (17) and Singh et al. (18) made notable strides in eliminating non-response bias using randomized scrambling techniques. Gupta et al. (19) developed an estimator for population variance based on Diana and Perri's randomization strategy (20). Earlier, Gupta et al. (21, 22) introduced generalized scrambling for quantitative optional models together with a unified measure of respondent privacy and model efficiency, which Lovig et al. (23) later extended to mixture binary designs. Saleem et al. (24) introduced refined estimators for population means within the randomized response framework. Zapata et al. (13) further enhanced Warner's classical method, while Azeem et al. (14) proposed a weighted measure that combines efficiency and privacy to assess randomized response methods' performance. Azeem et al. (25) subsequently developed a unified measure for evaluating the performance of randomized response techniques. Siddiqui et al. (26) took the analysis further by examining models incorporating variable correlations. Additional notable work by other researchers includes Azeem et al. (27), Bhat et al. (28), Gupta et al. (29), Jenum et al. (30), and Zahid et al. (31).

Recent literature relevant to this study can be categorized into three key areas. First, ongoing methodological advancements in randomized response techniques have improved privacy protection, robustness, and estimation accuracy. Contributions from Audu et al. (10), Saleem et al. (24), and Sayed et al. (32) introduced calibrated optional designs, comparative privacy–efficiency assessments, improved scrambling techniques, and models addressing evasive response bias. Hsieh et al. (5) showcased how indirect questioning could provide more reliable prevalence estimates for sensitive outbreak-related behaviors. Second, health survey methodologies have emphasized the considerable impact of privacy concerns, response bias, and survey design on data quality. Studies by Conduah et al. (4), Hendrix et al. (2), Soneson et al. (3), and von Glasenapp (6) highlighted how these factors influence the accuracy of health-related data. Third, applied public-health research from Pakistan has highlighted the marked variation in vaccine hesitancy, acceptance, and other health behaviors across different populations and settings. Studies by Tahir et al. (33), Kazmi et al. (34), Khalid et al. (8), Khan et al. (35), Khattak et al. (36), and Quaife et al. (37) underline the diversity in vaccine-related attitudes in urban, underserved, and nationally sampled populations. Beyond public health, self-report survey instruments are widely applied in other research domains, including educational and organizational studies (38) and applied engineering evaluation (39), where accurate measurement is equally essential. Recent contributions by Honghe et al. (40), Wanqu et al. (41), and Shah et al. (42) further extend this line of work on survey-based measurement of sensitive outcomes.Collectively, this literature stresses the importance of privacy-protective survey strategies that are statistically efficient and practically applicable in sensitive public-health studies.

These methodological innovations have proven particularly valuable during the pandemic, which underscored the necessity of collecting accurate, sensitive health data. Since its emergence in 2019, the outbreak has had profound global impacts, affecting both physical and mental health on a massive scale (43). It created a complex interaction between various health factors, with respiratory distress being a major symptom that often led to more severe complications, such as organ failure and death (9, 44, 45). Long-term effects reported globally include sleep disorders, olfactory dysfunction, ageusia, persistent chest tightness, hair loss, fatigue, and cognitive impairment (46). Studies indicate that a substantial proportion of outbreak survivors (9%–63%) continue to experience persistent symptoms (9, 11, 47–50), leading to higher healthcare costs than similar post-viral conditions. Privacy-preserving, stratified survey designs therefore offer a practical pathway for improving data quality in future public health surveillance of sensitive behaviors and outcomes. To demonstrate the feasibility and effectiveness of the stratified randomized response models (SRRMs), we implemented them using real data on sensitive public health variables.

**Figure 1:**
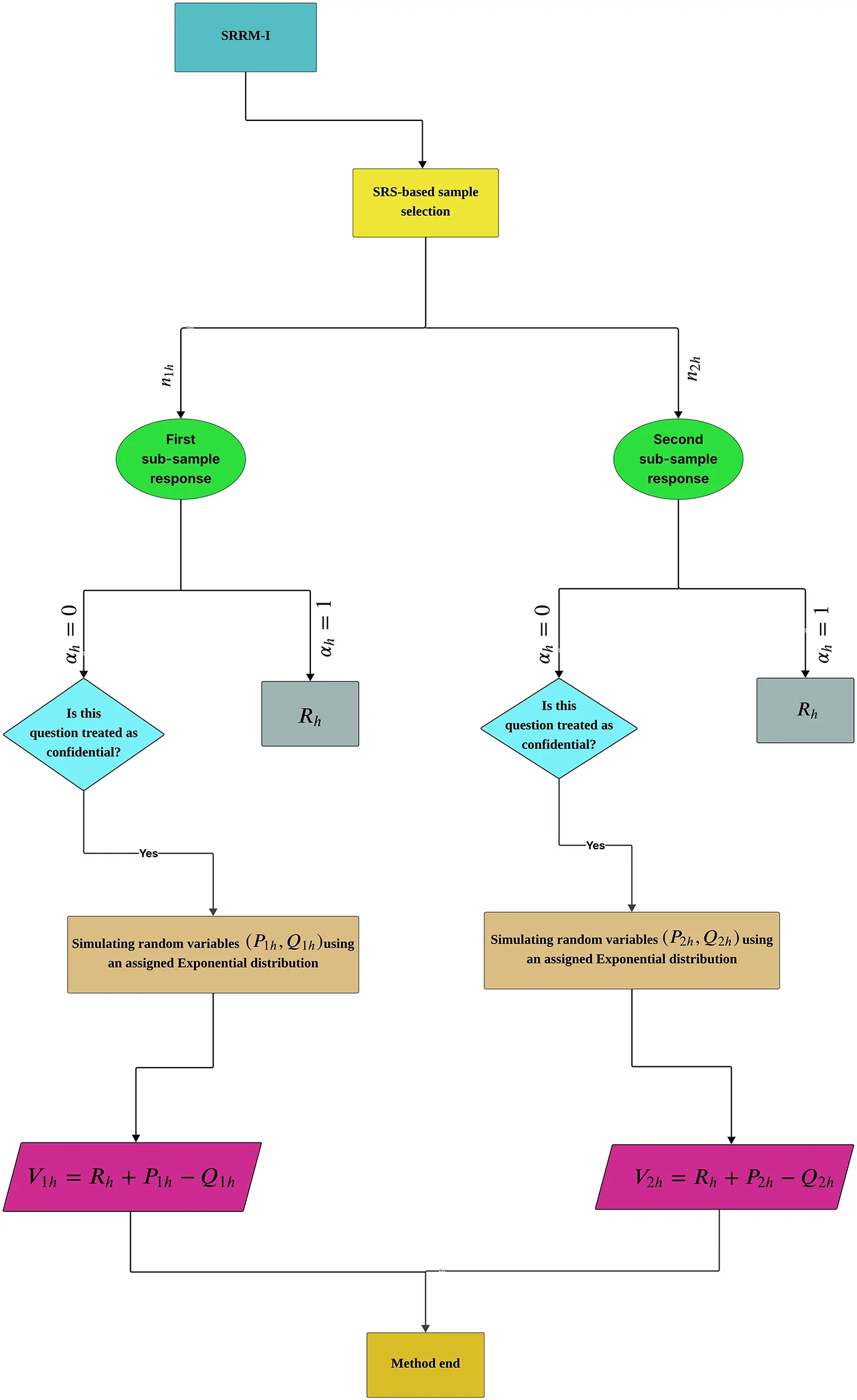
Operational structure of the single-stage SRRM-I framework. Two sub-samples are drawn from each stratum by SRSWR, and the true response *R*_*h*_ is masked by the simultaneous addition of *P*_*ih*_ and subtraction of *Q*_*ih*_, yielding the reported response *V*_*ih*_ = *R*_*h*_+*P*_*ih*_−*Q*_*ih*_. The design preserves respondent confidentiality while permitting unbiased estimation of the stratum mean and the sensitivity level.

**Figure 2:**
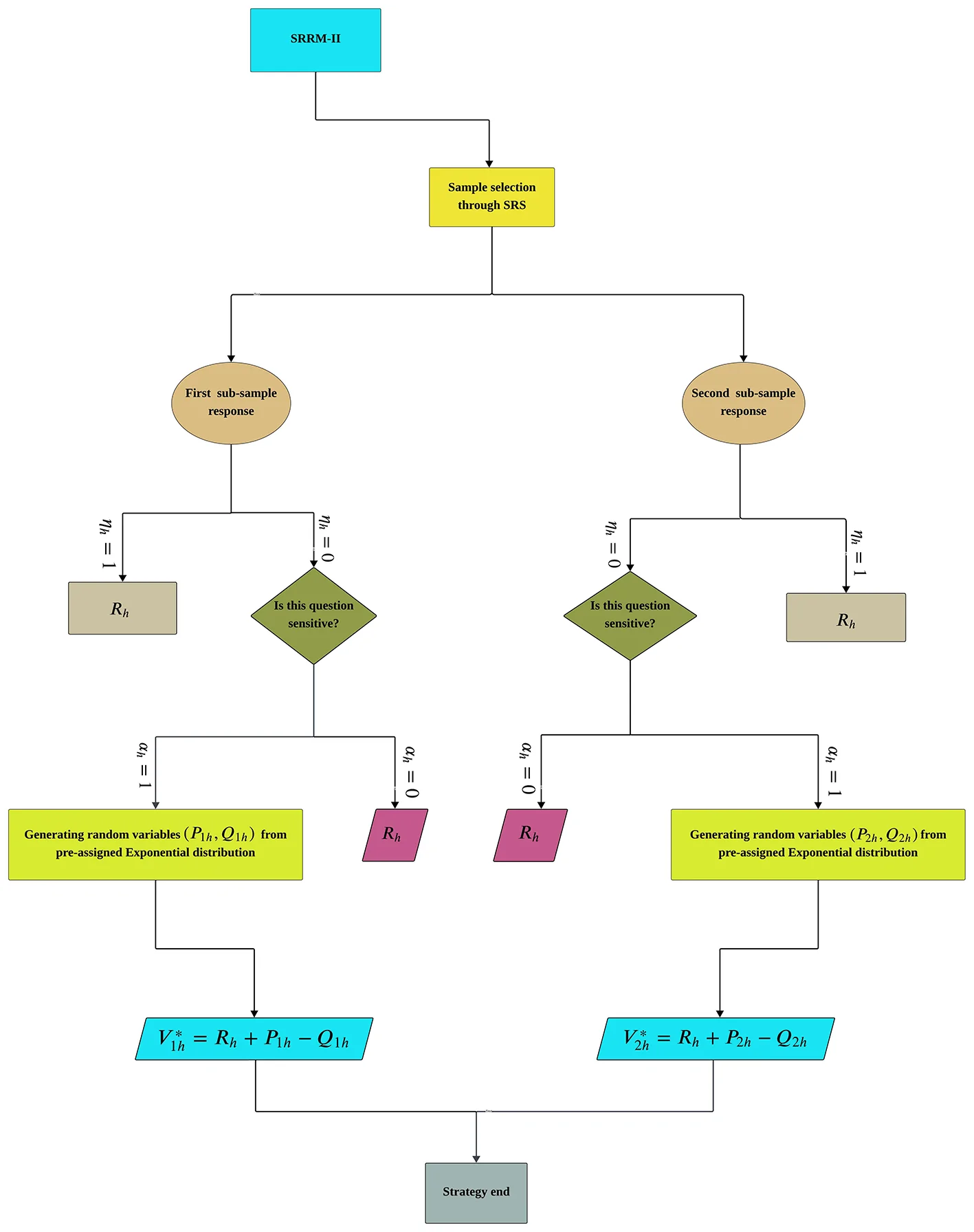
The skeletal mechanism underpinning SRRM-II through the two-stage randomized response process, in which a respondent reports the true value *R*_*h*_ with probability *T*_*h*_ or otherwise proceeds to the additive-subtractive scrambling stage.

Building on this background, the present study develops two privacy-preserving frameworks, SRRM-I and SRRM-II, and applies them to two sensitive public health outcomes: underreported outbreak cases and vaccine hesitancy. SRRM-I operates in a single stage, in which the true response of a respondent is masked simultaneously by an additive scrambling variable and a subtractive scrambling variable, so that confidentiality is preserved without losing the information required for unbiased estimation of the mean and the sensitivity level. SRRM-II extends this structure into two stages by introducing a truthful-reporting parameter, which allows a proportion of respondents to answer directly before the scrambling stage is applied, thereby providing additional flexibility in modeling disclosure behavior, particularly where the perceived sensitivity of the question is high. The operational structure of each framework, including sub-sample selection, the scrambling mechanism, and the form of the reported response, is presented in Figures 1, 2 of the Section 2. Both frameworks are applied within a stratified design covering urban and rural populations of Punjab, Pakistan, and their performance is compared with directly reported responses in terms of estimated prevalence, sensitivity level, and PRE.

## 2 Methods

The present section delineates the foundational theory and technique underlying the proposed additive and subtractive SRRMs. We established two SRRMs to estimate the mean μ_*k*_ and the sensitivity level *W*_*k*_, for *k* = I, II, of the corresponding stigmatizing variables. Under a stratified sampling context, the population is segmented into discrete strata, from which two distinct sub-samples of sizes *n*_1*h*_ and *n*_2*h*_ are extracted from each stratum to ensure uniform representation of every subgroup. Consequently, the split-sample methodology is regarded as the most dependable and advantageous approach in this situation. A primary cross-sectional survey carried out in the districts of Lahore, Faisalabad, and Sargodha in Punjab, Pakistan, between June and August 2021, provided the data used to support the study's conclusions.

Both study outcomes were treated as binary variables. COVID-19 infection status was coded as “yes” if at least one outbreak-related case occurred in the respondent's household or personally during June-August 2021, and “no” otherwise. Vaccine hesitancy was coded as “yes” if the respondent or any household member delayed or refused vaccination during the same period, and “no” otherwise. These outcomes were derived from the questionnaire, which specifically collected data on outbreak infection and vaccination behavior.

The questionnaire targeted two highly sensitive social determinants of health: self-reported outbreak infection status and reluctance to vaccinate, both of which are commonly underreported because of the social stigma attached to them. These fundamental public health measures were estimated using the split-sample scrambling procedure of the SRRMs, applied within a stratified sampling framework. Privacy was ensured through personalized randomization lists, immediate shredding of calculation forms, and anchored consent forms, all of which ensured that respondents remained anonymous. Pilot testing on a sample of 200 participants confirmed that a completion time of 15–20 min was feasible for each questionnaire, and aggregated respondent scores were in good agreement with the theoretical distributions, which confirmed the operational soundness of the models. All participants received standardized instructions before answering sensitive questions. They were informed that only aggregate results would be reported and that they should not reveal whether they had provided a true or a scrambled response. Respondents used pre-prepared random number tables to select values for *P*_*ih*_ and *Q*_*ih*_. Interviewers turned away during sensitive responses to maintain anonymity, and no personally identifiable information was recorded. Verbal informed consent was obtained from all participants.

The target sample size was determined before data collection using the standard formula for estimating a population proportion, $n_{0}=z^{2}p\left(1-p\right)/e^{2}$. Assuming a 95% confidence level (*z* = 1.96), maximum variability (*p* = 0.5), and a margin of error of *e* = 0.05, a minimum of 384 respondents was required for each of the urban and rural strata. This requirement was increased to 600 respondents per stratum in order to accommodate the split-sample design, in which each stratum is divided into two independent sub-samples that are analyzed separately, and to allow for anticipated non-response and for the loss of precision introduced by the scrambling mechanism. The resulting total of 1,200 participants was also consistent with the fieldwork capacity available during the June-August 2021 study period.

Within this total, 600 participants were drawn from urban areas (*n*_1_) and 600 from rural areas (*n*_2_). The weights for the two subgroups, *G*_1_ and *G*_2_, were initially calculated from the sample proportions, giving equal weights of 0.5 and reflecting their equal representation in the sample. However, to align the sample more closely with the actual population distribution and to mitigate potential bias, the weights were adjusted to *G*_1_ = 0.45 for the urban subgroup and *G*_2_ = 0.55 for the rural subgroup, accounting for the higher proportion of the rural population in the study area. These adjusted weights allow the estimates to reflect the population distribution more accurately, reducing bias arising from unequal representation of subgroups and improving the reliability of the resulting statistical inferences.

### 2.1 Sampling frame and participant selection

The sampling frame consisted of adult residents of selected urban and rural communities in Punjab, Pakistan, during the June-August 2021 study period. A stratified sampling design was used, with the population first divided into urban and rural strata and then into upper and lower subgroups based on area-level socioeconomic and residential characteristics, including population density, access to healthcare services, and local administrative classification. Eligible participants were adults aged 18 years or older, permanent residents of the selected areas, and willing to provide informed consent. Individuals below 18 years of age, temporary visitors, those unable to provide consent, and those unwilling to complete the questionnaire were excluded. Within each stratum, participants were selected using simple random sampling with replacement (SRSWR), ensuring that each eligible individual had an equal probability of selection at each sampling stage. Household or community lists were used where available; where complete lists were unavailable, systematic household selection was applied to approximate random selection while maintaining the planned stratum-specific allocation. Non-response was addressed by approaching additional eligible participants from the same stratum, and response status was documented. The final sample included 1,200 respondents, with 600 from urban areas and 600 from rural areas.

Within each stratum, participants were further divided into two equal sub-samples of 300 respondents each: upper urban (*n*_11_) and lower urban (*n*_21_) within the urban stratum, such that *n*_11_+*n*_21_ = *n*_1_, and upper rural (*n*_12_) and lower rural (*n*_22_) within the rural stratum, such that *n*_12_+*n*_22_ = *n*_2_. Randomized responses were then obtained in each sub-sample using the additive (*P*_*ih*_) and subtractive (*Q*_*ih*_) scrambling variables. This segmentation supports precise estimation by accounting for variation in reporting patterns, sensitivity levels, and socio-demographic characteristics across population subgroups.

### 2.2 Method design-I: privacy-preserving survey framework

In order to apply this conceptual framework, we first sketch the recommended response strategy, which is the principal mechanism supporting randomized but informative data collection under SRRM-I. Stratified designs are highly relevant in sensitive public health surveys because they can improve representativeness and allow subgroup-specific estimation while preserving overall precision. At the same time, stratification introduces additional design and estimation considerations when privacy-protecting mechanisms are applied. Existing studies using privacy-preserving survey methods in public health have provided valuable insights, but relatively few have focused on stratified frameworks tailored to heterogeneous populations and real-world field implementation in the context of outbreak misreporting and vaccine hesitancy (11, 47, 48). This gap is important because public health policy decisions often require disaggregated evidence, particularly where urban-rural differences shape exposure risk, health communication, and willingness to disclose sensitive information. The proposed approach employs a stratified random sampling design. The population of size *N* is segmented into *H* strata, each containing *N*_*h*_ individuals (*h* = 1, …, *H*), so that $\overset{H}{\underset{h=1}{\sum }}N_{h}=N$. The weight of each stratum is given by $G_{h}=\frac{N_{h}}{N}$.

As illustrated in Figure 1, the structural mechanism of SRRM-I during the randomized response process comprises sub-sample selection, the confidentiality decision, and the additive-subtractive scrambling step that produces the reported response. The SRRM-I technique uses SRSWR to select two distinct sample groups from each stratum (upper urban, lower urban, upper rural, and lower rural), with sample sizes *n*_1*h*_ and *n*_2*h*_ such that *n*_1*h*_+*n*_2*h*_ = *n*_*h*_.

The allocation of the sub-samples follows the proportional principle *n*_*ih*_ = *n*_*i*_*G*_*h*_, where *i* = 1, 2, and the overall sample sizes satisfy $\overset{H}{\underset{h=1}{\sum }}n_{1h}=n_{1}$ and $\overset{H}{\underset{h=1}{\sum }}n_{2h}=n_{2}$. In this context, *R*_*h*_ denotes the true answer in the *h*-th stratum, *P*_1*h*_ and *P*_2*h*_ denote the additive scrambling variables, *Q*_1*h*_ and *Q*_2*h*_ the subtractive scrambling variables, and α_*h*_ the indicator of whether a scrambled reply is provided within the *h*-th stratum, which occurs with probability *W*_*h*_. To mitigate response bias, the two random variables *P*_*ih*_ and *Q*_*ih*_ are generated for each respondent in the *i*-th sub-sample of stratum *h* from pre-defined Gamma distributions. The variable *P*_*ih*_ is added to the true answer *R*_*h*_, whereas *Q*_*ih*_ is subtracted. This strategy preserves confidentiality and mitigates bias while concealing the scrambling outcome from the interviewer, who nonetheless knows the means and variances of the randomization variables. The instructions for each participant in the *i*-th sub-sample of stratum *h* are therefore as follows.

- If the question is insensitive, report the true response *R*_*h*_;
- If the question is sensitive, report the scrambled response *R*_*h*_+*P*_*ih*_−*Q*_*ih*_.

This stratified sampling technique divides the population into homogeneous subgroups, each represented in proportion. Within each stratum, randomization minimizes response bias and the effects of social desirability, especially for sensitive questions. The distribution of the reported response for the *i*-th sub-sample of stratum *h* is obtained as


$$V_{ih}=\{\begin{array}{ll} R_{h} & \text{with likelihood}\;(1-W_{h}),\\ R_{h}+P_{ih}-Q_{ih} & \text{with likelihood}\;W_{h}. \end{array}$$

The combination of true and scrambled replies allows unbiased estimation of *R*_*h*_ and *W*_*h*_ while safeguarding respondent confidentiality. By integrating both response types, social desirability bias is reduced, providing more precise outcomes for sensitive questions. With the response mechanism defined, the next step is to construct unbiased and efficient estimators that infer the parameters of interest from the randomized responses. The SRRM-II design is introduced to address the limitations of the single-stage SRRM-I by incorporating a two-stage response mechanism with a customizable true-reporting probability, which offers additional flexibility for survey settings in which the perceived sensitivity of the question varies across subgroups or outcomes.

### 2.3 Method design-II: privacy-preserving survey framework

Building on the framework developed for SRRM-I, the SRRM-II model introduces a structured response mechanism that enhances respondent confidentiality and provides added flexibility in data collection. Following the same notation, a population of size *N* is divided among *H* strata, each of size *N*_*h*_ (*h* = 1, …, *H*), so that $\overset{H}{\underset{h=1}{\sum }}N_{h}=N$, and the relative weight of every stratum is $G_{h}=\frac{N_{h}}{N}$. Two distinct sub-samples are extracted within each stratum, with sample sizes *n*_1*h*_ and *n*_2*h*_, so that *n*_1*h*_+*n*_2*h*_ = *n*_*h*_. The allocation follows *n*_*ih*_ = *n*_*i*_*G*_*h*_, where *i* = 1, 2, and the aggregate sample sizes satisfy $\overset{H}{\underset{h=1}{\sum }}n_{1h}=n_{1}$ and $\overset{H}{\underset{h=1}{\sum }}n_{2h}=n_{2}$. We extend the single-stage design into the two-stage SRRM-II model in order to estimate the mean μ_*II*_ and the sensitivity level *W*_*II*_ of the stigmatizing variable *R*. In the *i*-th sub-sample of stratum *h*, each participant reports the true answer *R*_*h*_ with probability *T*_*h*_, or proceeds to stage two with probability 1−*T*_*h*_. In stage two, participants follow the SRRM-I procedure to generate two random variables, *P*_*ih*_ and *Q*_*ih*_, from pre-defined Exponential distributions; *P*_*ih*_ is added to the true answer, whereas *Q*_*ih*_ is subtracted. The instructions for each participant in the *i*-th sub-sample of stratum *h* are therefore as follows.

- In stage one, provide the true response *R*_*h*_ with probability *T*_*h*_; otherwise, proceed to stage two with probability 1−*T*_*h*_.
- In stage two, provide the true response *R*_*h*_ if the question is regarded as insensitive, and the scrambled response *R*_*h*_+*P*_*ih*_−*Q*_*ih*_ if the question is regarded as sensitive.

The distribution of the reported response $V_{ih}^{*}$ can therefore be stated as follows, describing the randomness and the reporting behavior of the replies within the *h*-th stratum.


$$V_{ih}^{*}=\{\begin{array}{ll} R_{h} & \text{with likelihood}\;T_{h}+(1-T_{h})(1-W_{h}),\\ R_{h}+P_{ih}-Q_{ih} & \text{with likelihood}\;(1-T_{h})W_{h}. \end{array}$$

This distribution allows respondents to provide either the true answer *R*_*h*_ or a scrambled reply *R*_*h*_+*P*_*ih*_−*Q*_*ih*_, depending on the perceived level of sensitivity. The probabilities *T*_*h*_ and *W*_*h*_ determine the chance that an individual reveals the true or the scrambled reply, which helps reduce bias while maintaining privacy. The reported values for the first sub-sample of size *n*_1*h*_ and for the second sub-sample of size *n*_2*h*_ in the *h*-th stratum are shown below.

Figure 2 presents the structural mechanism of SRRM-II, in which the truthful-reporting parameter *T*_*h*_ governs the transition between direct reporting and the scrambling stage of the randomized response process. In the SRRM-II technique, two independent sub-samples are chosen within each stratum (upper urban, lower urban, upper rural, and lower rural) through SRSWR, with sample sizes *n*_11_ and *n*_21_ in the urban stratum, for which *n*_11_+*n*_21_ = *n*_1_, and *n*_12_ and *n*_22_ in the rural stratum, for which *n*_12_+*n*_22_ = *n*_2_. In the *i*-th sub-sample of stratum *h*, each respondent either reports the true answer *R*_*h*_ with probability *T*_*h*_ or proceeds to stage two with probability 1−*T*_*h*_, where the randomization variables *P*_*ih*_ and *Q*_*ih*_ are generated from their pre-assigned distributions and applied additively and subtractively, as in the preceding technique.

Having specified both frameworks, we applied them to the survey data on sensitive public health variables. The following sections report the empirical implementation of SRRM-I and SRRM-II in assessing underreported outbreak cases and vaccine hesitancy in the Lahore, Faisalabad, and Sargodha districts of Punjab, Pakistan. Privacy was maintained through the secure handling of questionnaire responses, and participants were assured of their right to withdraw at any stage without consequence. The resulting estimates of the stratum means, sensitivity levels, and percentage relative efficiency (PRE) are presented in the Results section and interpreted in the Section 5, where they are compared with the directly reported values and with the benchmark model of Shahid et al. (11).

## 3 Results

Table 1 summarizes the mean, variance, and true-response parameters used for the urban and rural strata. The mean true responses were μ_*R*1_ = 8 in the urban stratum and μ_*R*2_ = 6 in the rural stratum, with variances $\sigma_{R1}^{2}=2$ and $\sigma_{R2}^{2}=3$, under the stratum weights *G*_1_ = 0.45 and *G*_2_ = 0.55 specified in the Section 2.

**Table 1:** Descriptive statistics of urban and rural subgroups, including scrambling parameters and true-response values.

Subgroups	Average measurements (overall)	Spread measurements (overall)	True-response parameters (overall)
Urban (subgroup-I)	μ_11_, μ_21_ (4, 4); λ_11_, λ_21_ (1, 1)	$\delta_{11}^{2}$, $\delta_{21}^{2}$ (1, 1); $\gamma_{11}^{2}$, $\gamma_{21}^{2}$ (3, 2)	μ_*R*1_ (8), $\sigma_{R1}^{2}$ (2)
Rural (subgroup-II)	μ_12_, μ_22_ (5, 4); λ_12_, λ_22_ (1, 1)	$\delta_{12}^{2}$, $\delta_{22}^{2}$ (2, 3); $\gamma_{12}^{2}$, $\gamma_{22}^{2}$ (3, 2)	μ_*R*2_ (6), $\sigma_{R2}^{2}$ (3)

Table 2 reports the directly reported and SRRM-I estimated outbreak infection rates for the two strata. In urban areas, the estimated rate was 15.3% (SE = 0.8; 95% CI: 13.73–16.87%), against a directly reported rate of 10.5%, at a sensitivity level of *W*_1_ = 0.60. In rural areas, the estimated rate was 16.7% (SE = 1.5; 95% CI: 13.76–19.64%), against a directly reported rate of 13.7%, at a sensitivity level of *W*_2_ = 0.40. The estimated rates therefore exceeded the directly reported rates by 4.8 percentage points in urban areas and by 3.0 percentage points in rural areas. The corresponding PRE values were *PRE*_μ_*I*__ = 248.7 for urban areas and 202.2 for rural areas, and *PRE*_*W*_*I*__ = 263.4 and 259.3 for the sensitivity-level estimates.

**Table 2:** Regional estimates of outbreak underreporting using SRRM-I in urban and rural communities of Punjab, Pakistan.

Variables	Urban (upper and lower urban)	Rural (upper and lower rural)
Sample size (overall)	600	600
Sensitivity level, *W*	0.60	0.40
Reported cases (overall)	10.5%	13.7%
Estimated mean of SRRM-I (${\hat{\mu }}_{I}$)	15.3 (± 0.8)	16.7 (± 1.5)
Estimated sensitivity level of SRRM-I (Ŵ_*I*_)	0.60	0.40
PRE of mean for SRRM-I (*PRE*_μ_*I*__)	248.7	202.2
PRE of sensitivity level for SRRM-I (*PRE*_*W*_*I*__)	263.4	259.3

Table 3 reports the corresponding estimates obtained under SRRM-II. The estimated infection rate was 17.4% (SE = 1.5; 95% CI: 14.46–20.34%) in urban areas and 19.5% (SE = 1.4; 95% CI: 16.76–22.24%) in rural areas, against directly reported rates of 10.5% and 13.7%, respectively, corresponding to differences of 6.9 and 5.8 percentage points. The truthful-reporting parameter was set at *T* = 0.20 for urban areas and *T* = 0.10 for rural areas, with sensitivity levels of *W* = 0.60 and *W* = 0.40. The PRE values were *PRE*_μ_*II*__ = 198.9 for urban areas and 218.5 for rural areas, and *PRE*_*W*_*II*__ = 215.4 and 224.3 for the sensitivity-level estimates.

**Table 3:** Regional estimates of outbreak underreporting using SRRM-II in urban and rural communities of Punjab, Pakistan.

Variables	Urban (upper and lower urban)	Rural (upper and lower rural)
Sample size (overall)	600	600
Sensitivity level, *W*	0.60	0.40
Truthful-reporting parameter, *T*	0.20	0.10
Reported cases (overall)	10.5%	13.7%
Estimated mean of SRRM-II (${\hat{\mu }}_{II}$)	17.4 (± 1.5)	19.5 (± 1.4)
Estimated sensitivity level of SRRM-II (Ŵ_*II*_)	0.60	0.40
PRE of mean for SRRM-II (*PRE*_μ_*II*__)	198.9	218.5
PRE of sensitivity level for SRRM-II (*PRE*_*W*_*II*__)	215.4	224.3

Table 4 compares the directly reported values with the SRRM-I and SRRM-II estimates at the outcome level. For underreported outbreak cases, the directly reported value was 14.1%, whereas the SRRM-I and SRRM-II estimates were 17.2% (SE = 1.2; 95% CI: 14.85–19.55%) and 20.9% (SE = 1.0; 95% CI: 18.94–22.86%), corresponding to differences of 3.1 and 6.8 percentage points. For vaccine hesitancy, the directly reported value was 26.3%, compared with 27.4% (SE = 1.8; 95% CI: 23.87–30.93%) under SRRM-I and 27.2% (SE = 1.5; 95% CI: 24.26–30.14%) under SRRM-II, corresponding to differences of 1.1 and 0.9 percentage points. The PRE values were 330.3 for underreported outbreak cases and 290.6 for vaccine hesitancy. The parameters were set at *W* = 0.60 and *T* = 0.20 for underreported outbreak cases and at *W* = 0.40 and *T* = 0.10 for vaccine hesitancy, reflecting the different levels of perceived sensitivity of the two outcomes.

**Table 4:** Comparison of directly reported values and estimates obtained using the proposed SRRM-I and SRRM-II privacy-preserving frameworks for outbreak underreporting and vaccine hesitancy in Punjab, Pakistan.

Variables	Reported value	SRRM-I estimate	SRRM-II estimate	*W*	*T*	PRE
Underreported outbreak cases	14.1%	17.2% (± 1.2)	20.9% (± 1.0)	0.60	0.20	330.3
Vaccine hesitancy	26.3%	27.4% (± 1.8)	27.2% (± 1.5)	0.40	0.10	290.6

The PRE values reported in Tables 2–4 were obtained by comparing the one-stage SRRM-I and the two-stage SRRM-II with the corresponding one-stage and two-stage benchmark models of Shahid et al. (11). Each PRE was computed as the ratio of the variance of the benchmark estimator to the variance of the SRRM estimator, expressed as a percentage, so that values above 100 indicate improved efficiency relative to the benchmark. All 95% confidence intervals were obtained as the point estimate ±1.96 standard errors.

**Figure 3:**
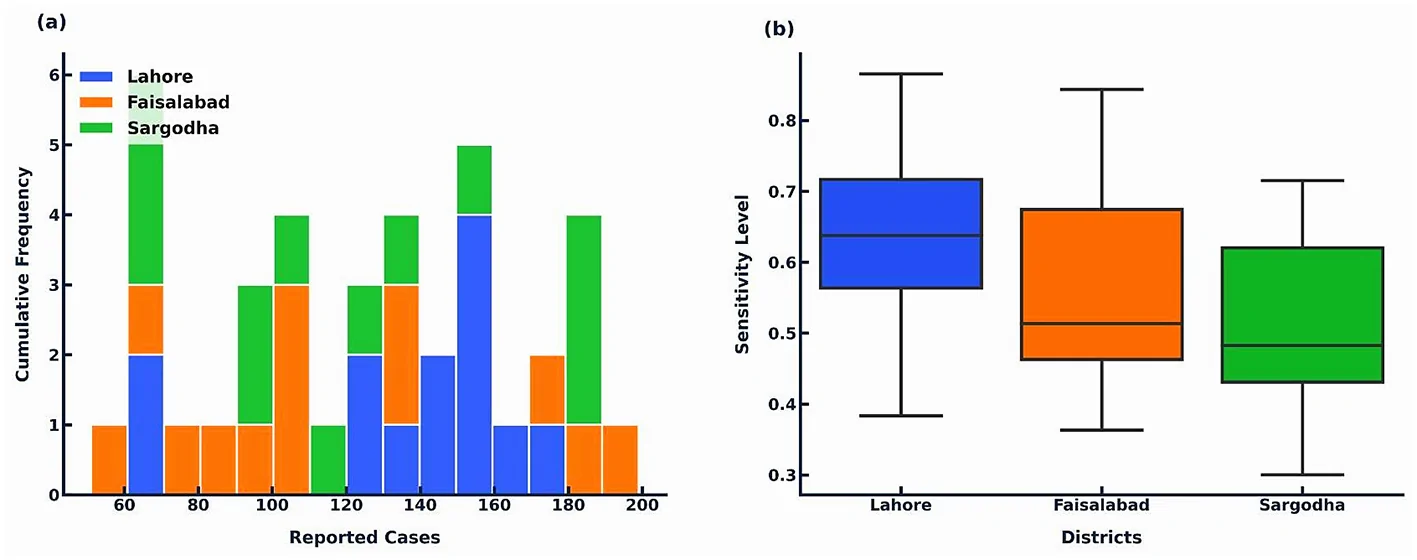
Distribution of self-reported outbreak cases and response sensitivity across the three survey districts. Panel **(a)** shows the cumulative frequency of self-reported cases among respondents in Lahore, Faisalabad, and Sargodha. Panel **(b)** shows the distribution of the respondent-level sensitivity levels in each district.

**Figure 4:**
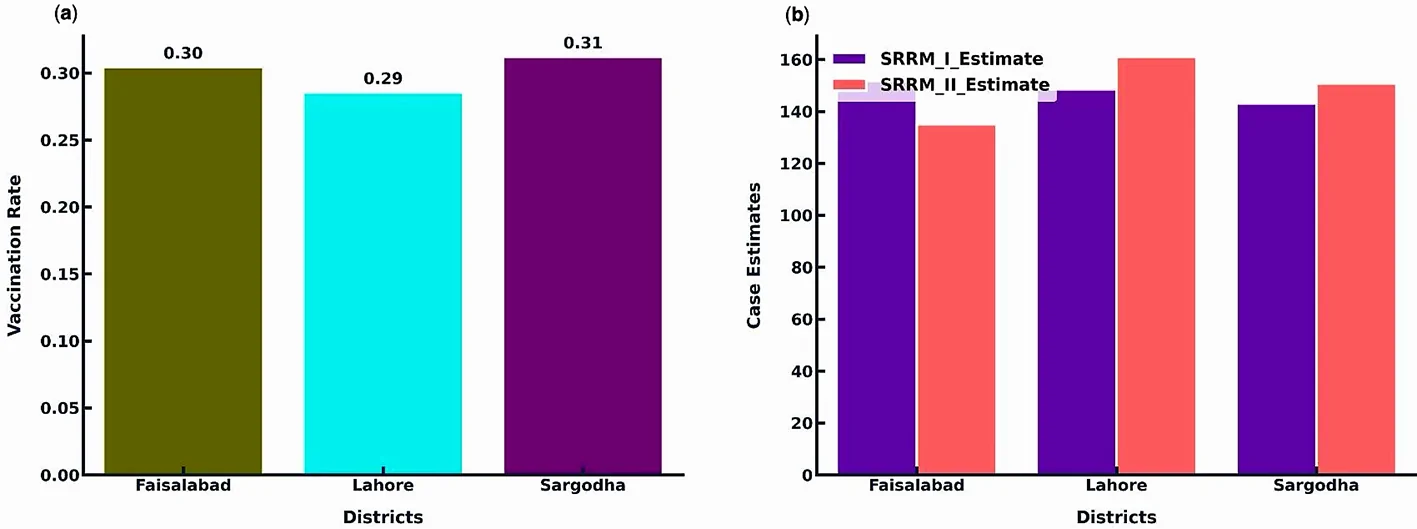
Self-reported vaccination coverage and SRRM-based estimates of self-reported outbreak cases in the three survey districts. Panel **(a)** shows the self-reported vaccination coverage for Faisalabad, Lahore, and Sargodha. Panel **(b)** compares the SRRM-I and SRRM-II case estimates for the same districts.

**Figure 5:**
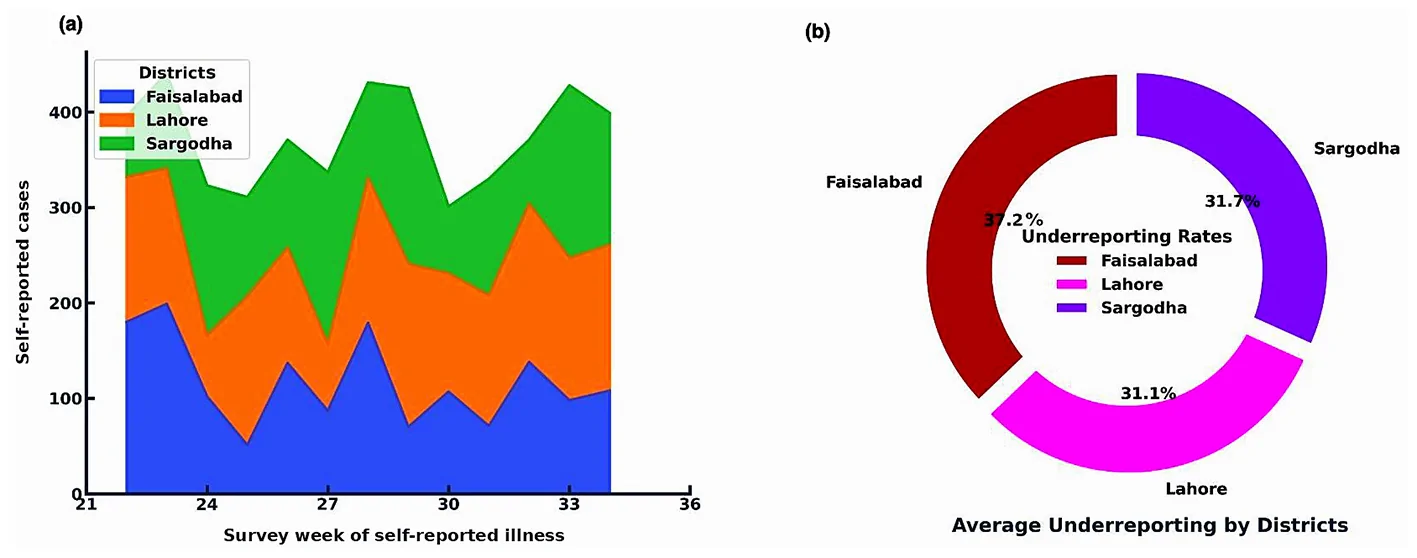
Self-reported cases by survey week and district-level underreporting. Panel **(a)** shows the number of self-reported cases by the survey week of illness reported by respondents in Faisalabad, Lahore, and Sargodha. Panel **(b)** shows the average underreporting for each district, expressed as the proportion of estimated cases not disclosed under direct questioning.

Figures 3–5 summarize the questionnaire responses collected in the three study districts, covering the distribution of self-reported outbreak cases, the sensitivity levels obtained for respondents, the self-reported vaccination coverage, the SRRM-I and SRRM-II case estimates, and the district-level underreporting obtained from the difference between the direct and the privacy-preserving responses

Figure 3a shows the cumulative frequency distribution of self-reported cases in Lahore, Faisalabad, and Sargodha. The distribution is dispersed across the full range of reported values rather than concentrated in any single district, indicating that respondents in all three districts reported a comparable spread of case counts. Figure 3b shows the distribution of the respondent-level sensitivity levels from which the stratum-level values of *W* reported in Tables 2, 3 were obtained. The median sensitivity level was highest in Lahore, intermediate in Faisalabad, and lowest in Sargodha, with interquartile ranges that overlap substantially across the three districts.

Figure 4a shows the self-reported vaccination coverage, which was closely similar in the three districts: 0.31 in Sargodha, 0.30 in Faisalabad, and 0.29 in Lahore. Figure 4b compares the SRRM-I and SRRM-II estimates of self-reported outbreak cases for the same districts. SRRM-II produced higher estimates than SRRM-I in Lahore and Sargodha, whereas in Faisalabad the SRRM-II estimate fell below the SRRM-I estimate.

Figure 5a shows the number of self-reported cases by the survey week to which respondents assigned their illness. The series fluctuates from week to week without a sustained upward or downward trend, and all three districts contribute throughout the recall period. Figure 5b shows the average underreporting for each district, expressed as the proportion of estimated cases that were not disclosed under direct questioning: 37.2% in Faisalabad, 31.7% in Sargodha, and 31.1% in Lahore.

## 4 Discussion

Both privacy-preserving frameworks produced estimates that exceeded the directly reported values for every outcome and in both strata. Since the two procedures differ from direct questioning only in the way the response is masked, the consistent upward difference is most plausibly attributable to non-disclosure under direct questioning rather than to the estimation procedure itself. The magnitude of the difference was substantially larger for underreported outbreak cases (3.1 and 6.8 percentage points at the outcome level) than for vaccine hesitancy (1.1 and 0.9 percentage points), which suggests that infection status carried a greater perceived disclosure risk than vaccination attitudes in this setting.

The two strata differed in both the level of the estimates and the assumed sensitivity of the question. Rural respondents recorded higher estimated infection rates than urban respondents under both frameworks (16.7% vs. 15.3% under SRRM-I and 19.5% vs. 17.4% under SRRM-II), whereas the discrepancy between reported and estimated values was larger in urban areas (4.8 and 6.9 percentage points) than in rural areas (3.0 and 5.8 percentage points). Differences in reporting patterns between the two groups may reflect contextual factors such as literacy, social exposure, healthcare access, or the degree of stigma attached to infection status; none of these factors was measured directly, and no causal inference can be drawn from a cross-sectional design. Because no inferential test was applied to the urban–rural contrast, these differences should be read as descriptive rather than as evidence of a statistically significant regional effect.

The comparison between the two frameworks is not uniform across outcomes. For underreported outbreak cases, SRRM-II produced markedly higher estimates than SRRM-I in both strata and at the outcome level, which is consistent with the expectation that the additional truthful-reporting stage recovers responses that a single scrambling stage leaves concealed. For vaccine hesitancy, however, the two frameworks produced almost identical estimates (27.4% and 27.2%), with substantially overlapping confidence intervals. The advantage of the two-stage design therefore appears greatest where perceived sensitivity is high and diminishes for outcomes that respondents are already relatively willing to disclose, so that the truthful-reporting parameter is best regarded as a design instrument to be matched to the sensitivity of the specific outcome rather than as a uniform improvement.

The choice of *T*_*h*_ reflected this reasoning. The values *T* = 0.20 for urban and *T* = 0.10 for rural respondents were selected on the basis of pilot testing and theoretical considerations: urban respondents were expected to be more familiar with survey procedures and therefore marginally more willing to answer directly, whereas the lower value applied in rural areas provides stronger protection where stigma and confidentiality concerns are more pronounced. These values balance the need for a proportion of truthful responses, which stabilizes the estimator, against the confidentiality that motivates the design.

The district-level results point in the same direction but are not ordered along a simple urban–rural axis. Underreporting was greatest in Faisalabad (37.2%) and almost identical in Sargodha (31.7%) and Lahore (31.1%), while the sensitivity level was highest in Lahore, where measured underreporting was lowest. Since all values derive from the same questionnaire administered under a common protocol, these district differences reflect variation in respondents' willingness to disclose and in the perceived sensitivity of the questions rather than differences in case ascertainment or in the coverage of any reporting system. District-level disclosure behavior should therefore be taken into account when a privacy-preserving instrument is designed, and the parameters *W* and *T* adjusted to the sensitivity of the outcome under study.

In terms of efficiency, all PRE values exceeded 100, indicating that both frameworks estimated the mean and the sensitivity level with smaller variance than the benchmark models of Shahid et al. (11). The gains were of a similar order across strata for SRRM-I (248.7 and 202.2 for the mean) and SRRM-II (198.9 and 218.5), and were larger at the outcome level (330.3 and 290.6), where the estimates pool both strata and therefore draw on the full sample. Taken together, the results indicate that the additional variance introduced by the scrambling mechanism is more than offset by the reduction in response bias, so that privacy protection is obtained without a loss of statistical precision.

Three features of the design constrain the interpretation of these findings. First, goodness-of-fit and model diagnostics for the scrambling distributions cannot be applied directly, because the observed values are scrambled rather than true responses; this is inherent to any randomized response framework, and the robustness of the approach is instead evidenced by the consistency of the estimated means, sensitivity levels, and PRE values across strata and outcomes. Second, the scrambling variables *P*_*ih*_ and *Q*_*ih*_ were calibrated from pilot testing and theoretical considerations rather than estimated from the field data, so the estimates depend on the adequacy of that calibration. Third, the reported comparisons between strata, districts, and outcomes are descriptive, and the differences observed indicate directions for further investigation rather than established effects.

## 5 Strengths and limitations

The utilization of SRRMs in this study strengthens the methodological basis of privacy-preserving data estimation, improving both the accuracy of survey data and the protection of respondent confidentiality. The split-sample design, which integrates additive and subtractive randomization within each sub-sample, facilitates data collection on sensitive public health topics such as the under-reporting of outbreak cases and vaccine hesitancy. By combining stratified random sampling with privacy-enhancing field protocols, the study supports consistent data collection across urban and rural populations and allows regional patterns to be described separately for each stratum. As shown by the PRE values, the approach also increases statistical efficiency relative to the benchmark models. More broadly, SRRMs have potential applications across various disciplines in the social sciences, particularly where sensitive information must be protected in order to maintain respondent trust.

Several limitations should nevertheless be acknowledged. Because the data were collected between June and August 2021, the empirical estimates should be interpreted as period-specific evidence from that survey period in Punjab, Pakistan, rather than as current prevalence estimates. This positions the study primarily as a methodological contribution: although the data reflect the 2021 context, the proposed privacy-preserving survey approach remains relevant for current and future surveillance systems where underreporting, stigma, social desirability bias, and confidentiality concerns continue to affect the accuracy of sensitive health data. In addition, since the study is based on a cross-sectional survey conducted in Punjab, Pakistan, the results may not be directly transferable to other populations with different socio-demographic characteristics. Finally, as noted in the Section 5, the comparisons between strata, districts, and outcomes are descriptive rather than inferential, and the estimates depend on the calibration of the scrambling variables.

## 6 Conclusion

This paper demonstrates the effectiveness of privacy-preserving SRRM frameworks in addressing the challenges of collecting precise and confidential data on sensitive public health outcomes. These frameworks strike an effective balance between maintaining respondent privacy and producing reliable estimates, helping to reduce response biases, including social desirability bias, for sensitive outcomes such as underreported outbreak cases and vaccine hesitancy. The empirical findings indicate that both frameworks improved estimation efficiency and privacy protection compared with directly reported responses. The advantage of the two-stage design was, however, most pronounced for the more sensitive outcome, indicating that the truthful-reporting parameter should be matched to the perceived sensitivity of the outcome under study. The practical application of these frameworks in Punjab, Pakistan, highlights their utility in public health research, where privacy concerns often hinder accurate reporting, and their potential contribution to surveillance and decision-making based on more reliable evidence on sensitive health outcomes. Because the procedures rely only on pre-printed random number tables, they can be implemented in routine household surveys without specialized devices, software, or additional interviewer training. Future work should extend the frameworks to longitudinal and multi-country designs and evaluate them against directly validated records, so that the recovery of concealed responses can be assessed inferentially rather than descriptively. Overall, the proposed SRRM frameworks offer an ethical and statistically useful approach for collecting sensitive public health data, and their application in Punjab supports their potential use in other regions facing similar privacy and data-collection challenges.

## Data Availability

The raw data supporting the conclusions of this article will be made available by the authors, without undue reservation.
